# Machine Learning Assisted Prediction of Power Conversion Efficiency of All-Small Molecule Organic Solar Cells: A Data Visualization and Statistical Analysis

**DOI:** 10.3390/molecules27185905

**Published:** 2022-09-11

**Authors:** Norah Alwadai, Salah Ud-Din Khan, Zainab Mufarreh Elqahtani, Shahab Ud-Din Khan

**Affiliations:** 1 Department of Physics, Collega of Sciences, Princess Nourah bint Abdulrahman University, P.O. Box 84428, Riyadh 11671, Saudi Arabia; 2 Sustainable Energy Technologies Center, College of Engineering, King Saud University, P.O. Box 800, Riyadh 11421, Saudi Arabi; 3 Pakistan Tokamak Plasma Research Institute (PTPRI), Islamabad P.O. Box 3329, Pakistan

**Keywords:** small molecule donors, machine learning, Pearson correlation, random forest regressor

## Abstract

Organic solar cells are famous for their cheap solution processing. Their industrialization needs fast designing of efficient materials. For this purpose, testing of large number of materials is necessary. Machine learning is a better option due to cheaper prediction of power conversion efficiencies. In the present work, machine learning was used to predict power conversion efficiencies. Experimental data were collected from the literature to feed the machine learning models. A detailed data visualization analysis was performed to study the trends of the dataset. The relationship between descriptors and power conversion efficiency was quantitatively determined by Pearson correlations. The importance of features was also determined using feature importance analysis. More than 10 machine learning models were tried to find better models. Only the two best models (random forest regressor and bagging regressor) were selected for further analysis. The prediction ability of these models was high. The coefficient of determination (R2) values for the random forest regressor and bagging regressor models were 0.892 and 0.887, respectively. The Shapley additive explanation (SHAP) method was used to identify the impact of descriptors on the output of models.

## 1. Introduction

The recent development of society is the result of technological advancement that is the fruit of great scientific research [1,2,3]. Extensive research is going on in the field of material science [4,5,6]. High industrialization has led to many environmental issues [7,8]. Therefore, clean energy is an essential need of modern society. Solar energy is a huge source of energy. One of the most promising ways to gather and process solar energy is photovoltaic (PV) devices [9]. Third-generation solar cells are referred to as emerging technologies [10]. Their performance efficiencies are high. Among all these emerging technologies, organic solar cells (OSCs) have drawn considerable attention from academic and industrial communities due to their peculiar characteristics, such as ease of use, sustainability, adjustability, compactness, as well as transparency compared to conventional silicon-based inorganic solar cells. The primary process of photovoltaic cells is the absorption of sunlight harvested by the active layers. Organic molecules are structurally π-conjugated molecules with alternating π and σ bonds [11,12,13]. Thus, they are composed of discontinuous energy levels naming HOMO and LUMO levels that are an abbreviation of the highest occupied molecular orbital and the lowest unoccupied molecular orbitals, respectively. The difference between these two energy levels is known as the band gap.

Bulk-junction deposition, in which donor and accepter materials are mixed thoroughly, is the famous type of photoactive layer structure [14,15]. Co-deposition of the two materials improves the closeness of contact between the two comparable semiconductors, which is the basis for this type of organic solar cell [16,17].

Scharber’s model has been used to predict the performance of organic solar cells [18]. Different, less realistic assumptions are used to derive this model. This makes it less accurate [19]. Only electronic parameters of materials used in active layers are used to predict power conversion efficiency (PCE). It is difficult to include other descriptors such as structure, topology, and thermodynamics. Therefore, there are fewer chances to enhance its performance.

In recent years, machine learning (ML) has gained fame in material science [20,21]. Machine learning is much faster than density functional theory and molecular dynamics simulations [22,23]. The increase in computer power and development of efficient software have enhanced the potential of machine learning. It is can be used for discovery, data mining, prediction, and design of new materials [24,25,26]. Compared to traditional computational and experimental approaches, machine learning has developed quickly [27,28,29].

In the current work, machine learning-based regression models were trained to predict the PCE of all-small molecule organic solar cells. Multiple models were trained and the best models were selected for further analysis. Their parameters were tuned. A detailed data visualization analysis was also performed to find the hidden trends of data. Pearson correlation was used to find the relationship between parameters and power conversion efficiency. The feature importance of parameters in training of models was also calculated. The Shapley additive explanation (SHAP) method was used to identify the impact of parameters on the output of models.

## 2. Results and Discussion

The performance of different materials depends on their chemistry [30,31]. Chemical data can help to understand their behavior [32,33]. The hidden patterns of data can provide much useful information [34,35].

### 2.1. Visualization Analysis of Data

**Figure 1:**
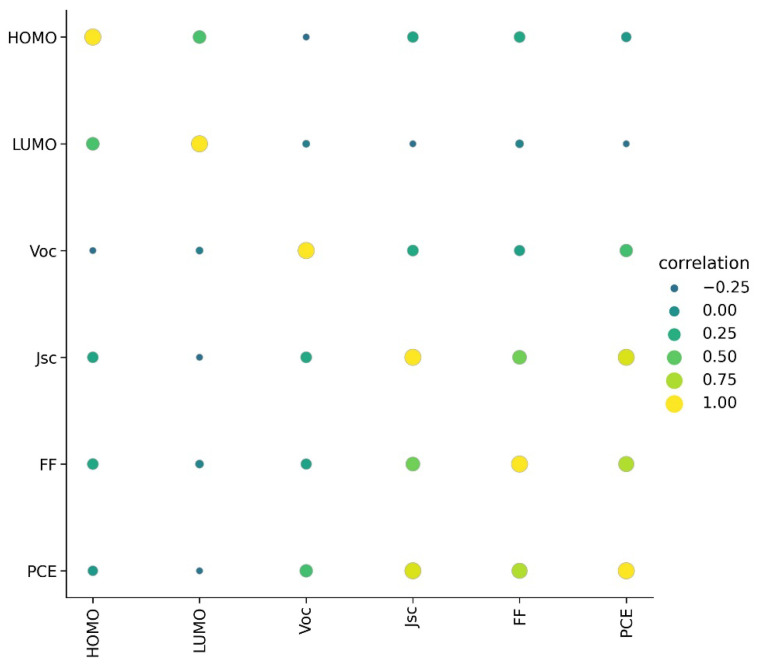
Correlation between parameters in dataset.

A detailed visualization analysis of data was performed. A heat map of correlation between PCE and other parameters is given in Figure 1. Only Jsc showed a high positive correlation with PCE; HOMO showed very low correlation with PCE and LUMO showed very low negative correlation with PCE. This indicates relatively less dependence of PCE on the energy level of donor materials.

**Figure 2:**
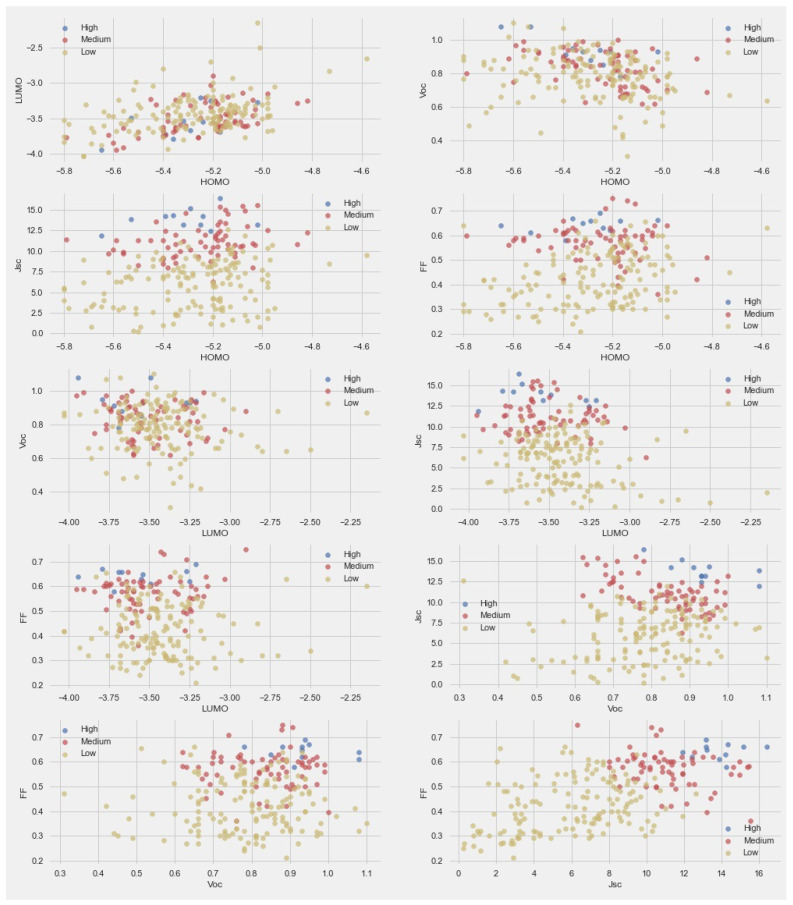
Pair-scatter plot between the parameters in dataset.

To better understand the data and effect of various parameters on PCE, we classified the PCE into three categories—high: PCE > 7, medium: PCE > 4, and low: PCE < 4. The paired scatter plots are given in Figure 2. For the scatter plot comparison between LUMO and HOMO, the trend of PCE was mixed. This means energy levels did not have a significant effect on PCE. The scatter plot comparison between Voc and HOMO indicated a high PCE at higher Voc values and middle HOMO values. The scatter plot of HOMO with Voc and FF did not provide any clear trend. The scatter plots of LUMO with Voc and FF indicated that a higher PCE was found at lower LUMO values.

**Figure 3:**
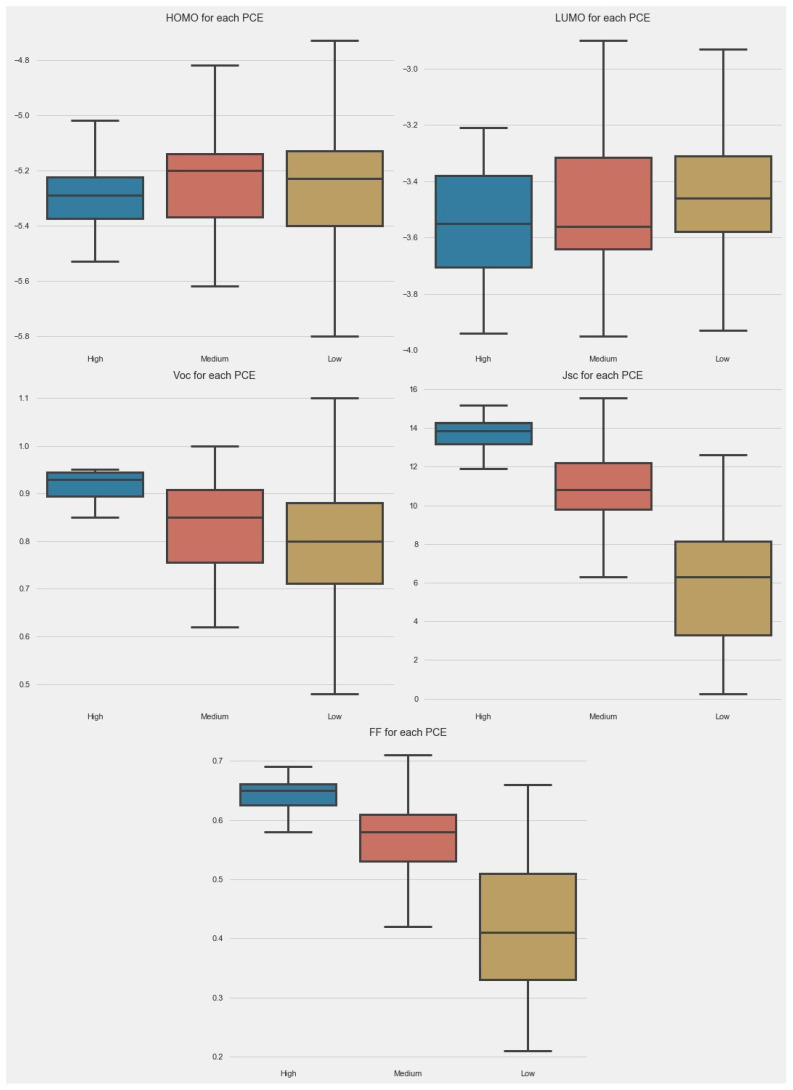
Box plot of parameters in dataset.

The box plot allowed us to look at the data in another way. The box plots for different parameters are given in Figure 3. In the case of HOMO, the majority of PCE points were boxed between −5.1 and −5.4 eV. The small-size box for a high PCE indicates that the control of the HOMO level of donors can help to achieve a high PCE. In the case of LUMO, the size of boxes was almost the same in all categories. In the cases of Jsc, Voc, and FF, the size of boxes was very small for a high PCE.

### 2.2. Correlation Analysis of Descriptors with PCE

The calculated molecular descriptors were used as input for model training. Molecular descriptors represent the chemistry of donor molecules. It is an open secret that the PCE of organic solar cells significantly depends on the chemical structure of materials that are used for OSCs [36,37]. Molecular descriptors present the chemical features of materials in numerical form [38,39].

**Figure 4:**
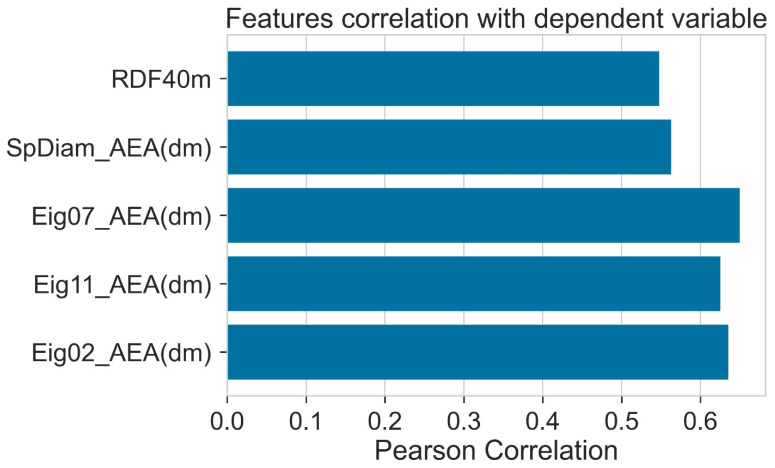
Pearson correlation of features with dependent variable (PCE).

The correlation of different descriptors with the PCE is given in Figure 4. Eig07_AEA (dm) showed a high positive correlation with PCE. Correlation of all the descriptors with PCE was higher than 0.5. The details of descriptors are given in Table 1.

### 2.3. Feature Importance

**Figure 5:**
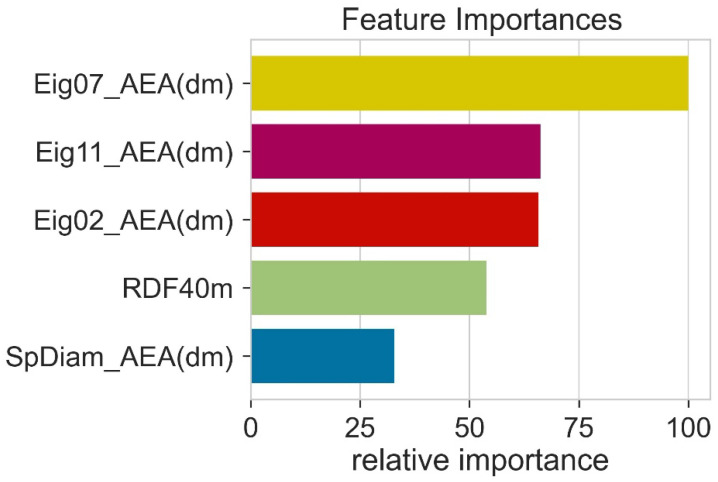
The relative importance of various features in machine learning models.

During model training, all the features (descriptors) do not play an equal role in model performance. Therefore, it is necessary to determine the relative importance of different features. The feature importance was calculated using random forest. The feature importance was obtained by computing the reduced training loss when using this feature. Higher feature importance values indicate that during model training, this feature has contributed more to the machine learning algorithm. This means that features with high feature importance values are helpful for machine learning model predictions. Eig07_AEA (dm) had high importance and SpDiam_AEA (dm) had less importance (Figure 5). However, the trend in Pearson correlation and feature importance was a little bit different. We further reduced the number of features; this decrease in feature numbers decreased the performance of machine learning models.

### 2.4. Shapley Additive exPlanations

**Figure 6:**
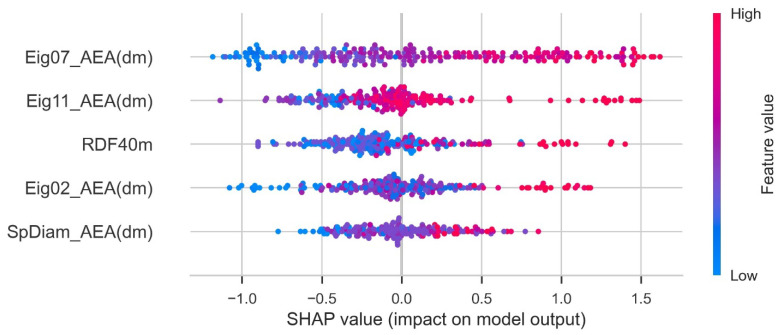
SHAP plot for parameters.

The Shapley additive exPlanations (SHAP) feature importance value was computed using the shap_values function provided by Python shap. This is a feature attribution method that connects the Shapley value and local interpretable model-agnostic explanations. The Shapley value, which is the basis of SHAP feature importance, is calculated using the average change in predicted values according to the presence or absence of the feature when considering all possible combinations of features. A large change in the predicted values depending on the presence or absence of a feature indicates that the corresponding feature significantly contributes to the training of the predictive ML model. It tells whether contribution of a feature is positive or negative. A higher value indicates the higher contribution to PCE. Each dot represents one sample point. The SHAP plot is given in Figure 6. Eig07_AEA (dm) had a strong impact on the output of the ML model.

### 2.5. Regression Analysis

**Figure 7:**
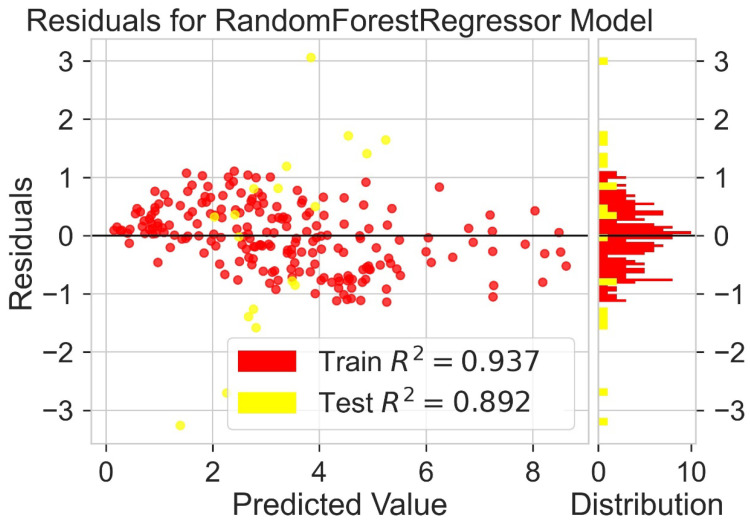
Residuals for random forest regressor model.

**Figure 8:**
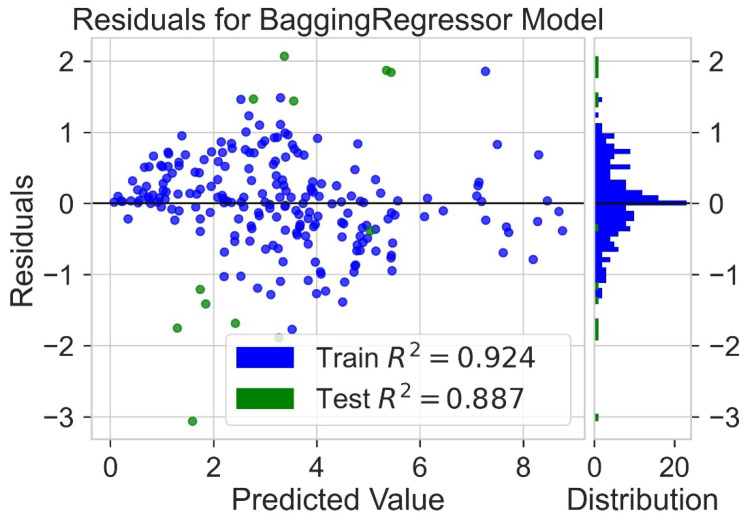
Residuals for bagging regressor model.

**Figure 9:**
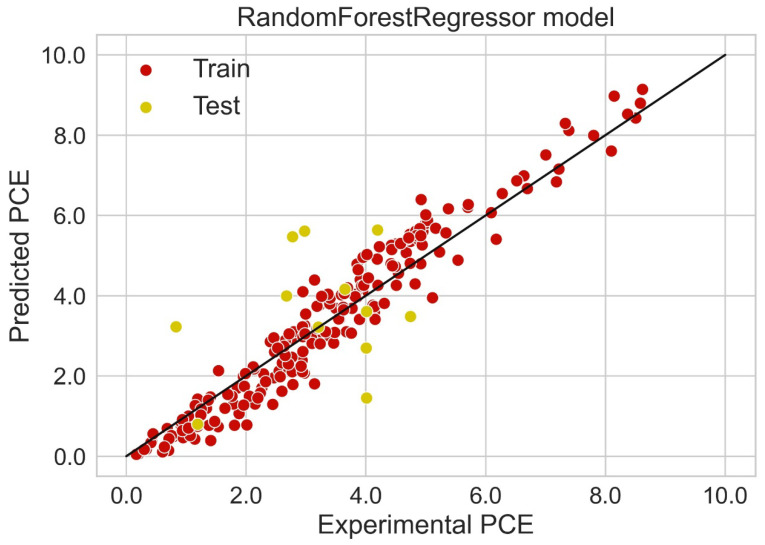
Scatter plot comparing experimental PCE and predicted PCE (random forest model).

**Figure 10:**
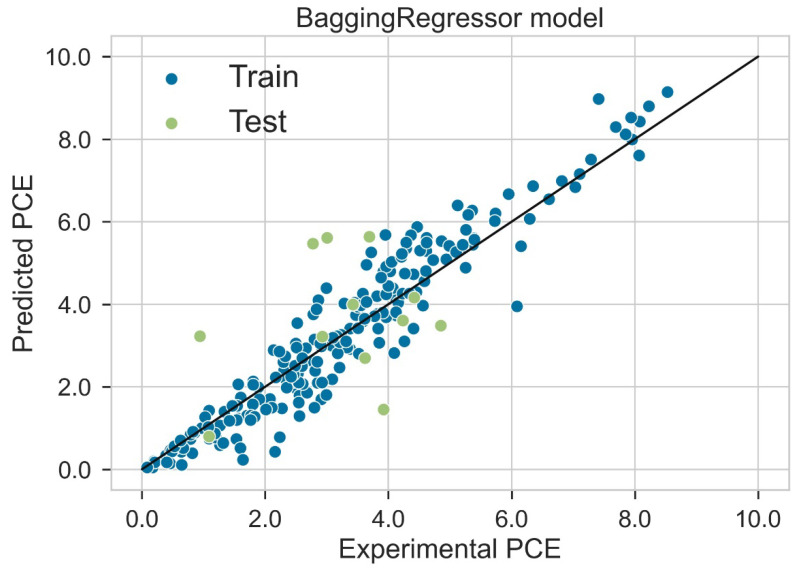
Scatter plot comparing experimental PCE and predicted PCE (bagging model).

Classification categorizes given data points into predefined groups. The wider the range of a group, the higher will be the classification accuracy. With the help of classification machine learning, it is possible to predict in which group the PCE of a particular donor will fall. In order to predict the PCE value of a particular donor, regression analysis was performed. More than ten regressors were used. The coefficient of determination (R^2^) values for the test set are given in Table 2. Random forest regressor and bagging regressor were the best models. These models were used for further analysis. Residuals of the best models were plotted. Basically, a residual plot is a plot that presents the residuals on the vertical axis and target variable on the horizontal axis. Residual value indicates the deviation of predicted values from actual values. The further the data point is away from zero, the more the predicted values will differ from actual values. The residual plot for the random forest model is given in Figure 7. In most cases, residual values were not very high. The distribution plot indicated major peaks near to zero. The residuals for the bagging model are given in Figure 8. The behavior of the bagging regressor was very similar to that of the random forest regressor. Both models were accurate enough; R^2^ values near 1 are considered good. The accurate prediction of different chemical properties can decrease dependence on expensive experimental methods [40,41,42]. The scatter plot comparing experimental PCE and predicted (random forest model) PCE is given in Figure 9. Most values were at the lower range. The scatter plot comparing experimental PCE and predicted (bagging model) PCE is given in Figure 10.

The random forest model was validated using an external set of data that was not used for training and testing purposes. Obtained results are given in Table 3. The low dissimilarity between predicted and experimental PCE values indicates that this model was reasonably accurate. An easy and fast prediction of PCE can speed the design of better donor materials.

A better understanding of chemical structure of materials helps to find better materials [46,47,48,49]. Our proposed model can help to predict the PCE quickly without any experimentation. Indeed, the performance prediction ability of machine leaning can be further improved by design-specific descriptors. It is well-known that the principle on which organic solar cells works is very complicated. The PCE of OSCs depends on a variety of factors [50,51]. Film morphology is one of them. The results from film morphology characterization can be explored using deep learning. Therefore, widespread research is needed to effectively utilize deep learning to understand the thin film morphological topographies of all-small molecule organic solar cells.

## 3. Methodology

### 3.1. Dataset

Our dataset had about 220 data points that were collected from research articles. Dataset is given in supporting information (Table S1). It contained the data of organic solar cells that were based on small molecule donors and fullerene acceptors. The dataset contained the HOMO and LUMO of donor materials as well as open-circuit voltage (V_OC_), short-circuit current density (J_SC_), and fill factor (FF) of solar cell devices. In research articles, the highest and average values of photovoltaic parameters are reported. We have selected the highest values. It is not easy to collect experimental data. The quality and volume of data strongly control the prediction ability of machine learning models.

### 3.2. Descriptors Calculation and Selection

About 3000 molecular descriptors were calculated using Dragon software [52]. Molecule descriptors are easy to calculate: a large number of descriptors can be calculated in a short time. As the number of descriptors was large, every descriptor was not important for model training. We have reduced their numbers in different ways. Descriptors with zero values were not chosen. Descriptors with the same values for all donors cannot provide any discriminating effect; therefore, they were removed. Many pairs of descriptors are similar, so in model training their role will be the same, and the use of both will not affect the performance of the model. So, one of the pair of descriptors was neglected.

### 3.3. Machine Learning

Machine learning was performed using the Scikit-learn Python library. This library provides many machine learning models to test. Data were handled using Pandas software. The calculated descriptors and target property (PCE) were placed in comma-separated values (.CSV) files. We tested more than 10 machine learning models. Two high-performing models were chosen for next step analysis. Their parameters were tuned to obtain better performance. Results from machine learning models were plotted using Seaborn and Matplotlib.

## 4. Conclusions

In this work, a sufficient amount of data from experimental sources was collected to train machine learning models, which can predict power conversion efficiencies. The accuracy of optimized of machine learning models was reasonably high. Pearson correlation analysis provided information about important parameters that play a critical role in PCE prediction. Eig07_AEA (dm) showed the highest correlation with power conversion efficiency. Its role was the greatest in model training. Multiple machine learning models were tried. The random forest model and bagging model were the best models with coefficient of determination (R2) values of 0.892 and 0.887, respectively. This approach can help to select better materials. The findings of our study suggest that machine learning methods provide a way forward for data visualization and performance prediction, which will speed up the industrial implementation of OSCs.

## Figures and Tables

**Table 1:** Detail of selected molecular descriptors.

No.	Name	Category	Description
1	RDF40m	RDF descriptor	Radial distribution function-040/weighted by relative mass
2	SpDiam_AEA (dm)	Edge adjacency indices	Spectral diameter from augmented edge adjacency mat. weighted by dipole moment
3	Eig07_AEA (dm)	Edge adjacency indices	Eigenvalue n. 7 from augmented edge adjacency mat. weighted by dipole moment
4	Eig11_AEA (dm)	Edge adjacency indices	Eigenvalue n. 11 from augmented edge adjacency mat. weighted by dipole moment
5	Eig02_AEA (dm)	Edge adjacency indices	Eigenvalue n. 2 from augmented edge adjacency mat. weighted by dipole moment

**Table 2:** Performance of various machine learning models (R^2^ for test set).

No	Model	R^2^
1	Random Forest Regressor	0.892
2	Bagging Regressor	0.887
3	Gradient Boosting Regressor	0.774
4	Light Gradient Boosting Machine	0.769
5	Extreme Gradient Boosting	0.667
6	Extra Trees Regressor	0.664
7	Support Vector Machine	0.632
8	Ridge Regression	0.607
9	K Neighbors Regressor	0.598
10	CatBoost Regressor	0.592
11	Linear Regression	0.588

**Table 3:** Validation of random forest model using external dataset.

Donor	Experimental PCE (%)	Predicted PCE (%)	Difference	Reference
DPP2T-3	2.48	2.81	0.33	[43]
DPP2T-4	3.30	2.98	0.32	[43]
DPP2T-5	1.90	2.13	0.23	[43]
DPPT	1.88	2.16	0.28	[44]
DPPSE	2.30	2.50	0.20	[44]
DPPTT	1.25	1.56	0.31	[44]
FDPP	4.32	4.71	0.39	[45]
CDPP	1.00	1.25	0.25	[45]

## Data Availability

Associated data used in this report are given in supporting information.

## Supplementary Materials

The following supporting information can be downloaded at: https://www.mdpi.com/article/10.3390/molecules27185905/s1, Table S1: Data of organic solar cells based on small molecule donor and fullerene acceptors.
