# Supplementary material for: Machine Learning Assisted Prediction of Power Conversion Efficiency of All-Small Molecule Organic Solar Cells: A Data Visualization and Statistical Analysis
Source: Molecules. 2022 Sep 11;27(18):5905. doi: 10.3390/molecules27185905 (PMC9502131; doi:10.3390/molecules27185905)
Supplement: Supplementary file 1 [file molecules-27-05905-s001.zip › molecules-1894592-supplementary.pdf]

<u>Supplementary Materials:</u>

Machine Learning Assisted Prediction of Power Conversion Efficiency of All-Small Molecule Organic Solar Cells: A Data Visualization and Statistical Analysis

Norah Alwadai [1,*], Salah Ud-Din Khan [2,*], Zainab Mufarreh Elqahtani [1] and Shahab Ud-Din Khan [3]

[1] Department of Physics, Collega of Sciences, Princess Nourah Bint Abdulrahman University, P.O. Box 84428, Riyadh 11671, Saudi Arabia
[2] Sustainable Energy Technologies Center, College of Engineering, King Saud University, P.O. Box 800, Riyadh 11421, Saudi Arabia
[3] Pakistan Tokamak Plasma Research Institute (PTPRI), P.O. Box 3329, Islamabad, Pakistan
* Correspondence: nmalwadai@pnu.edu.sa (N.A.); drskhan@ksu.edu.sa (S.U.-D.K.)

Table S1. Data of organic solar cells based on small molecule donor and fullerene acceptors

No	Donor Name	Acceptor Name	HOMO(eV)	LUMO(eV	Voc(V)	Jsc	FF(%)	PCE(%)	DOI of paper
1	DMPh (DPP-Py)2	PC71BM	-5.14	-3.45	0.68	14.5	0.55	5.47	10.1016/j.solener.2017.12.042
2	CP4	PC61BM	-5.41	-3.64	0.96	8.95	0.43	3.74	10.1016/j.solener.2019.11.073
3	SM1	PC71BM	-5.32	-3.16	0.99	11.18	0.56	6.2	10.1016/j.dyepig.2017.11.048
4	DTTFB(TDPP-T)2	PC71BM	-5.17	-3.63	0.696	12.44	0.595	5.15	10.1016/j.dyepig.2018.05.067
5	DTTFB(IID-T)2	PC71BM	-5.5	-3.69	0.756	9.44	0.447	3.19	10.1016/j.dyepig.2018.05.067
6	An(DTDPPA)2	PC61BM	-5.18	-3.62	0.905	11.32	0.51	5.26	10.1016/j.dyepig.2018.07.002
7	CP1	PC61BM	-5.4	-3.77	1.07	6.7	0.41	2.94	10.1016/j.dyepig.2019.107661
8	ZnPTDPPCN	PC61BM	-5.18	-3.68	0.82	12.12	0.43	4.26	10.1016/j.dyepig.2020.108211
9	ZnPTDPPO	PC61BM	-5.02	-3.57	0.76	15.54	0.36	4.24	10.1016/j.dyepig.2020.108211
10	BTI(2T-DCV-Hex)3	PC71BM	-5.36	-3.3	0.94	7.67	0.53	3.81	10.1016/j.dyepig.2020.108523
11	BTI(2T-CNA-EHex)3	PC71BM	-5.3	-3.25	0.94	6.8	0.53	3.41	10.1016/j.dyepig.2020.108523
12	N(Ph-2T-DCV-Hex)3	PC71BM	-5.34	-3.34	0.99	8.03	0.45	3.59	10.1016/j.dyepig.2020.108523
13	?u(3TIN)2	PC71BM	-5.18	-3.61	1	13.2	0.395	5.22	10.1016/j.dyepig.2020.108709

14	D(CATBTzT)BDT	PC61BM	-5.13	-3.31	0.93	5.99	0.64	3.61	10.1016/j.orgel.2013.11.034
15	DCAO3TBDT	PC61BM	-5.04	-3.24	0.95	8	0.6	4.56	10.1021/ja306865z
16	DR3TBDT	PC71BM	-5.02	-3.27	0.93	13.17	0.663	8.12	10.1021/ja403318y
17	DTTz-DTBTT	PC61BM	-5.05	-3.07	0.66	5.11	0.483	1.58	10.1039/c3nj01457f
18	DCAO3T(BDT)3T	PC61BM	-5.11	-3.54	0.93	9.77	0.599	5.44	10.1002/adma.201102790
19	DCAEH5TBT	PC61BM	-4.98	-3.21	0.78	7.1	0.554	3.07	10.1016/j.orgel.2013.03.006
20	BDT-3T-CA	PC61BM	-5.2	-2.9	0.88	6.3	0.75	4.16	10.1002/aenm.201300626
21	TIBDT	PC71BM	-5.2	-3.3	0.89	7.36	0.602	3.94	10.1016/j.dyepig.2014.08.027
22	TBDTCNR	PC61BM	-5.4	-3.63	0.9	9.08	0.66	5.42	10.1021/am4021928
23	C-IDT3T	PC71BM	-5.18	-3.29	0.91	10.52	0.496	4.75	10.1021/am501316y
24	R-IDT3T	PC71BM	-5.19	-3.27	0.9	11.55	0.49	5.09	10.1021/am501316y
25	PorODPP	PC61BM	-5.09	-3.55	0.81	10.52	0.5	4.26	10.1016/j.orgel.2015.11.035
26	PorSeDPP	PC61BM	-5.07	-3.61	0.71	14.93	0.547	5.81	10.1016/j.orgel.2015.11.035
27	BT3	PC71BM	-5.44	-3.45	0.92	8.2	0.5	3.77	10.1016/j.orgel.2015.01.032
28	BT4	PC71BM	-5.35	-3.67	0.84	10.82	0.58	5.27	10.1016/j.orgel.2015.01.032
29	BDT(CDTRH)2	PC71BM	-5.38	-3.54	0.94	10.42	0.62	6.07	10.1039/C4TA05807K
30	1	PCBM	-5.4	-2.8	0.64	1	0.32	0.2	10.1039/c0cc01229g
31	2	PCBM	-5.5	-3.3	0.78	0.26	0.25	0.05	10.1039/c0cc01229g
32	3	PCBM	-5.2	-3.1	0.98	3.41	0.31	1	10.1039/c0cc01229g
33	BTZ-P6t	PCBM	-5.52	-3.09	0.66	0.28	0.27	0.05	10.1021/ja910420t
34	TBTZ-P6t	PCBM	-5.33	-3.21	0.89	2.9	0.21	0.56	10.1021/ja910420t
35	BTZT-P6t	PCBM	-5.49	-3.35	0.45	0.8	0.3	0.11	10.1021/ja910420t
36	A-P6t	PCBM	-5.51	-2.98	0.89	3.1	0.45	1.27	10.1021/ja910420t
37	PA-P6t	PCBM	-5.44	-3.04	0.95	2.57	0.48	1.2	10.1021/ja910420t
38	A-P6d	PCBM	-5.01	-2.5	0.65	0.76	0.34	0.17	10.1021/ja910420t
39	PA-P6d	PCBM	-5.21	-2.7	0.88	1.16	0.32	0.34	10.1021/ja910420t
40	DPP(TNa)2	PC61BM	-5.18	-3.47	0.84	4.06	0.509	2.61	10.1016/j.dyepig.2013.09.022
41	DPP(TFNa)2	PC61BM	-5.2	-3.56	0.92	4.18	0.544	3.15	10.1016/j.dyepig.2013.09.022
42	DMM-TPA-Th2-MMN	PC71BM	-4.86	-3.28	0.89	10.8	0.42	4.04	10.1016/j.solmat.2013.03.020
43	TPA-Th2-MMN	PC71BM	-4.98	-3.28	0.89	10.36	0.34	3.05	10.1016/j.solmat.2013.03.020
44	TPAâ€“DPP	PC71BM	-4.98	-3.48	0.69	1.86	0.39	0.39	10.1016/j.orgel.2014.03.011
45	TPAâ€“DPPâ€“P	PC71BM	-4.98	-3.46	0.73	5.59	0.37	1.5	10.1016/j.orgel.2014.03.011
46	TPA(DPPâ€“P)2	PC71BM	-5.03	-3.45	0.78	9.24	0.48	3.42	10.1016/j.orgel.2014.03.011

47	1	PC71BM	-5.6	-3.47	1.1	3.2	0.35	1.2	10.1021/ol500809e
48	2	PC71BM	-5.33	-3.45	0.95	5.8	0.47	2.6	10.1021/ol500809e
49	H4FT6F	PCBM	-4.95	-3.05	0.7	3.03	0.37	0.77	10.1039/c0nj00045k
50	5	PC61BM	-5.02	-2.15	0.87	2.03	0.6	1.06	10.1002/adfm.200901827
51	7a	PCBM	-5.16	-3.24	0.44	1.08	0.31	0.15	10.1002/ange.200903125
52	5a	PCBM	-5.25	-3.42	0.63	3.28	0.36	0.74	10.1002/ange.200903125
53	5b	PCBM	-5.27	-3.45	0.66	3.49	0.37	0.84	10.1002/ange.200903125
54	5c	PCBM	-5.16	-3.18	0.42	2.79	0.42	0.49	10.1002/ange.200903125
55	5d	PCBM	-5.14	-3.37	0.31	12.6	0.47	1.79	10.1002/ange.200903125
56	5e	PCBM	-5.17	-3.37	0.54	3	0.39	0.64	10.1002/ange.200903125
57	MD344	PCBM	-5.71	-3.31	0.57	2.8	0.28	0.44	10.1039/b813341g
58	MD352	PCBM	-5.58	-3.24	0.63	2.9	0.27	0.49	10.1039/b813341g
59	MD334	PCBM	-5.78	-3.58	0.49	3.1	0.29	0.43	10.1039/b813341g
60	ATOP4	PCBM	-5.64	-3.4	0.73	4	0.32	0.91	10.1039/b813341g
61	IDOP301	PCBM	-5.8	-3.52	0.77	4	0.29	0.87	10.1039/b813341g
62	MD376	PCBM	-5.8	-3.76	0.9	5.3	0.32	1.54	10.1039/b813341g
63	MD304	PCBM	-5.59	-3.68	0.76	6.3	0.36	1.74	10.1039/b813341g
64	AS1	PC61BM	-5.56	-3.39	0.9	3.15	0.43	1.23	10.3390/molecules201219798
65	AS2	PC61BM	-5.38	-3.45	0.88	8.01	0.58	4.1	10.3390/molecules201219798
66	EH-DPP-TFPV	PCBM	-5.13	-3.46	0.86	3.3	0.5	1.4	10.1039/c1ee01544c
67	BTD3	PC71BM	-5.56	-3.64	0.94	9.84	0.58	5.36	10.1039/C6CP00243A
68	BTD4	PC71BM	-5.5	-3.78	0.92	11.28	0.6	6.02	10.1039/C6CP00243A
69	V-BDT	PC71BM	-5.34	-3.64	0.89	6.88	0.61	3.73	10.1016/j.orgel.2017.01.041
70	TH-Q	PC61BM	-5.12	-2.93	0.74	1.63	0.3	0.36	10.1021/am5027538
71	TQ5	PC71BM	-5.69	-3.64	0.83	0.79	0.26	0.17	10.1021/acsami.5b09460
72	BTTR	PC71BM	-5.32	-3.54	0.93	13.2	0.65	8	10.1016/j.orgel.2017.12.003
73	9RFL	PC61BM	-5.43	-3.5	0.718	9.91	0.3	2.13	10.1016/j.optmat.2019.04.001
74	SBDT1	PC71BM	-5.08	-3.46	0.78	9.97	0.51	3.99	10.1016/j.cclet.2019.07.018
75	SBDT3	PC71BM	-5.33	-3.59	0.89	9.96	0.5	4.45	10.1016/j.cclet.2019.07.018
76	STYTYT	PC71BM	-5.26	-3.77	0.97	12.54	0.508	6.17	10.1016/j.orgel.2018.02.039
77	BDP-dBDT	PC71BM	-5.06	-3.6	0.7	12.98	0.62	5.61	10.1016/j.orgel.2018.05.049
78	SM1	PC71BM	-5.27	-3.27	0.81	9.38	0.52	3.95	10.1016/j.orgel.2018.11.024
79	SM2	PC71BM	-5.23	-3.21	0.93	11.94	0.59	6.55	10.1016/j.orgel.2018.11.024
80	SM3	PC71BM	-5.21	-3.25	0.93	12.42	0.62	7.16	10.1016/j.orgel.2018.11.024

81	Porph-BT-DPA	PC71BM	-5.33	-3.52	0.72	7.48	0.39	2.11	10.1016/j.orgel.2019.03.012
82	Porph-BT-BDT	PC71BM	-5.53	-3.53	0.83	6.65	0.38	2.07	10.1016/j.orgel.2019.03.012
83	TQ7	PC71BM	-5.64	-3.6	0.83	4.89	0.29	1.18	10.1021/acsami.5b09460
84	TQ9	PC71BM	-5.57	-3.57	0.81	6.28	0.37	1.86	10.1021/acsami.5b09460
85	SM1	PC71BM	-5.33	-3.26	0.76	6.14	0.54	2.5	10.1039/C5TA09023G
86	SM2	PC71BM	-5.29	-3.32	0.84	10.18	0.57	4.91	10.1039/C5TA09023G
87	SM3	PC71BM	-5.38	-3.93	0.86	7.04	0.35	2.11	10.1039/C5TA09023G
88	1a	PC61BM	-5.2	-3	0.76	6.05	0.33	1.54	10.1016/j.orgel.2012.08.023
89	SM1	PC71BM	-5.18	-3.55	0.86	9	0.39	3	10.1021/am500522x
90	SM2	PC71BM	-5.19	-3.59	0.91	8.37	0.49	3.71	10.1021/am500522x
91	SM3	PC71BM	-5.22	-3.49	0.92	7.23	0.37	2.47	10.1021/am500522x
92	a,a-DH6TDPP	PC61BM	-4.73	-2.83	0.67	8.42	0.45	2.33	10.1021/jp8031572
93	DPP(CT)2	PC71BM	-5.35	-3.24	0.94	8.55	0.5	4.02	10.1039/c2ta00148a
94	CNDPP	PC71BM	-5.17	-3.5	0.72	13.6	0.476	4.73	10.1002/adma.201302848
95	DPP-DTF	PC71BM	-4.82	-3.25	0.69	12.24	0.51	4.3	10.1039/c5cc07435e
96	DPP-TP6	PC71BM	-5.5	-3.69	0.93	8.27	0.54	4.2	10.1021/cm401244x
97	CSDPP2	PC70BM	-5.22	-3.18	0.78	8.06	0.52	3.23	10.1016/j.orgel.2014.05.033
98	CSDPP4	PC70BM	-5.08	-3.14	0.72	10.63	0.6	4.65	10.1016/j.orgel.2014.05.033
99	CSDPP1	PC70BM	-5.14	-3.2	0.82	10.82	0.56	4.96	10.1039/c3ra44926b
100	BTCN-O	PC71BM	-5.59	-3.95	0.969	11.34	0.59	6.27	10.1021/acs.chemmater.9b01278
101	MV71	PC71BM	-5.24	-3.55	0.85	14.21	0.63	7.61	10.1021/acsaem.8b00024
102	MV72	PC71BM	-5.29	-3.67	0.88	15.16	0.66	8.8	10.1021/acsaem.8b00024
103	TB-BDT6T	PC61BM	-5.31	-3.63	0.85	6.87	0.59	3.41	10.1021/acsaem.8b00415
104	TS-BDT6T	PC61BM	-5.4	-3.67	0.96	8.47	0.61	4.89	10.1021/acsaem.8b00415
105	TT-BDT6T	PC61BM	-5.35	-3.64	0.97	9.4	0.62	5.64	10.1021/acsaem.8b00415
106	2FDRCN5T	PC71BM	-5.65	-3.94	1.08	11.9	0.64	8.3	10.1021/acsaem.8b02020
107	(Rh-TiC-T)2-DFBT	PC71BM	-5.45	-3.23	0.87	11.34	0.55	5.41	10.1021/acsaem.9b00303
108	DRBDT-TVT	PC71BM	-5.11	-3.41	0.879	10.73	0.73	6.87	10.1021/acsami.7b17961
109	DRBDT-STVT	PC71BM	-5.14	-3.43	0.907	10.25	0.74	6.84	10.1021/acsami.7b17961
110	P3T4-VCN	PC71BM	-5.68	-3.69	0.88	3.52	0.42	1.29	10.1021/acsami.8b13928

111	P3T4-INCN	PC71BM	-5.56	-3.91	0.99	9.66	0.59	5.68	10.1021/acsami.8b13928
112	C2T2BTF2	PC71BM	-5.15	-3.57	0.66	8.89	0.55	3.23	10.1021/acssuschemeng.9b07600
113	1	PC71BM	-5.21	-3.34	0.95	8.73	0.58	4.81	10.1039/c7cp08308d
114	M1	PC71BM	-5.21	-3.03	0.77	9.88	0.63	4.79	10.1039/c7cp08653a
115	CSDPP3	PC70BM	-5.28	-3.24	0.9	7.34	0.46	3.04	10.1039/c3ra44926b
116	CSDPP9	PC71BM	-5.62	-3.46	1.02	7.38	0.38	2.86	10.1039/C5RA16812K
117	CSDPP10	PC71BM	-5.51	-3.46	0.94	7.68	0.4	2.89	10.1039/C5RA16812K
118	CSDPP11	PC71BM	-5.4	-3.4	0.88	9.18	0.44	3.55	10.1039/C5RA16812K
119	CSDPP12	PC71BM	-5.54	-3.52	0.94	8.12	0.4	3.05	10.1039/C5RA16812K
120	TPATDPP	PC61BM	-5.28	-3.38	0.97	7.07	0.3	2.06	10.1039/C4CC04020A
121	A-AMIDE	PC71BM	-5.33	-3.47	0.82	11	0.41	3.65	10.1021/cm503915t
122	A-ESTER	PC71BM	-5.28	-3.57	0.84	3.9	0.44	1.45	10.1021/cm503915t
123	DPP(TBTH)2	PC71BM	-5.19	-3.47	0.76	5.7	0.33	1.43	10.1002/adfm.201201599
124	DPP(TBIND)2	PC71BM	-5.07	-3.33	0.81	4.31	0.3	1.03	10.1002/adfm.201201599
125	BTF	C60	-5.65	-3.88	0.66	3.22	0.32	0.75	10.1016/j.dyepig.2012.03.021
126	BFF	C60	-5.69	-3.86	0.6	3.3	0.39	0.8	10.1016/j.dyepig.2012.03.021
127	1	PC71BM	-5.2	-3.4	0.73	4.3	0.31	1.3	10.1002/adma.201103177
128	2	PC71BM	-5.2	-3.5	0.81	6.2	0.3	1.7	10.1002/adma.201103177
129	3	PC71BM	-5.3	-3.2	0.73	3.2	0.29	0.7	10.1002/adma.201103177
130	4a	PC71BM	-5.2	-3.2	0.77	5.7	0.55	2.7	10.1002/adma.201103177
131	BO-DPP-BTZ	PC70BM	-5.25	-3.5	0.78	2.4	0.48	0.89	10.1039/c3cp52929k
132	DPP-DBF	PC71BM	-5.36	-3.56	0.78	1.15	0.24	0.23	10.1016/j.matchemphys.2014.06.048
133	DPP-ACN	PC71BM	-5.35	-3.54	0.81	2.25	0.27	0.52	10.1016/j.matchemphys.2014.06.048
134	HTTBDB	PC71BM	-5.13	-3.58	0.64	9.66	0.46	2.81	10.1039/c3cp54548b
135	1	PC71BM	-5.31	-3.6	0.63	14.6	0.58	5.3	10.1002/aenm.201300240
136	DPPBI	PC61BM	-5.32	-3.61	0.86	7.46	0.37	2.25	10.1016/j.dyepig.2016.03.033
137	DPPBIT	PC71BM	-5.2	-3.26	0.8	8.8	0.4	2.7	10.1039/C5TA00367A
138	DPPBIT4F	PC71BM	-5.23	-3.27	0.74	10.5	0.71	5.4	10.1039/C5TA00367A
139	OD-DPP-A-PY	PC70BM	-5.72	-4.03	0.87	6.2	0.419	1.96	10.1016/j.orgel.2013.05.035
140	HD-DPP-A-PY	PC70BM	-5.72	-4.03	0.85	8.89	0.417	2.95	10.1016/j.orgel.2013.05.035

141	OD-DPP-PY	PC70BM	-5.51	-3.73	0.79	2.38	0.272	0.46	10.1016/j.orgel.2013.05.035
142	SPDPP	PC71BM	-4.98	-3.68	0.92	2.79	0.27	0.69	10.1016/j.solmat.2011.05.006
143	TPDPP	PC71BM	-5.17	-3.51	0.77	3.92	0.57	1.71	10.1016/j.solmat.2011.05.006
144	SM1	PC61BM	-5.1	-3.35	0.88	8.62	0.36	2.74	10.1039/c3tc32214a
145	SM2	PC61BM	-5.07	-3.37	0.84	11.9	0.38	3.76	10.1039/c3tc32214a
146	SM3	PC61BM	-5.14	-3.4	0.93	10.3	0.32	3.1	10.1039/c3tc32214a
147	SM4	PC61BM	-5.12	-3.41	0.9	9.73	0.33	2.92	10.1039/c3tc32214a
148	M1	PC61BM	-5	-3.31	0.49	6.6	0.46	1.48	10.1039/C5RA01946J
149	M2	PC61BM	-5.17	-3.39	0.84	7.67	0.31	1.99	10.1039/C5RA01946J
150	M3	PC61BM	-5.13	-3.48	0.89	7.17	0.35	2.23	10.1039/C5RA01946J
151	M4	PC61BM	-5.26	-3.51	0.98	9.04	0.35	3.1	10.1039/C5RA01946J
152	DT-DPP(AAnAT)2	PC61BM	-5.26	-3.77	0.77	9.28	0.614	4.39	10.1002/ajoc.201500068
153	An-2	PC61BM	-5.36	-3.69	0.87	9.24	0.63	5.07	10.1039/C5TC03844H
154	NDT(TDPP)2	PC61BM	-5.4	-3.68	0.84	11.27	0.42	4.06	10.1021/ja202791n
155	(TDPP)2	PC71BM	-5.19	-3.66	0.48	7.4	0.37	2.05	10.1016/j.orgel.2012.09.004
156	T(TDPP)2	PC71BM	-5.17	-3.68	0.8	4.3	0.43	1.3	10.1016/j.orgel.2012.09.004
157	ph(TDPP)2	PC71BM	-5.31	-3.65	0.93	9.09	0.47	3.88	10.1016/j.orgel.2012.09.004
158	T2(TDPP)2	PC71BM	-5.14	-3.55	0.78	6.8	0.57	3	10.1016/j.orgel.2013.03.031
159	TT(TDPP)2	PC71BM	-5.11	-3.56	0.81	9.3	0.53	4	10.1016/j.orgel.2013.03.031
160	(PH2(TDPP)2	PC71BM	-5.21	-3.57	0.86	8.3	0.53	3.8	10.1016/j.orgel.2013.03.031
161	NPT(TDPP)2	PC71BM	-5.18	-3.58	0.87	9.5	0.53	4.4	10.1016/j.orgel.2013.03.031
162	BDT(TDPPT)2	PC71BM	-5.3	-3.44	0.76	5.22	0.55	2.19	10.1039/c3nr03048b
163	DPP2AN(9,10)	PC71BM	-5.38	-3.35	0.97	4.09	0.317	1.45	10.1021/acsami.5b03338
164	DPP2AN(2,6)	PC71BM	-5.19	-3.25	0.82	11.9	0.554	5.44	10.1021/acsami.5b03338
165	CZ(TDPP)2	PC61BM	-5.03	-3.32	0.66	4.12	0.44	1.5	10.1039/c2nj40963a
166	FL(TDPP)2	PC61BM	-5.16	-3.36	0.66	3.17	0.3	0.78	10.1039/c2nj40963a
167	8	PC61BM	-5.46	-3.64	0.718	6.67	0.356	1.7	10.1021/cm301095x
168	BT(TDPPTTT_2	PC71BM	-5.07	-3.77	0.511	2.2	0.655	0.74	10.1039/c3ee24351f
169	F(TDPP(TTT)2	PC71BM	-4.98	-3.36	0.78	6.2	0.518	2.51	10.1039/c3ee24351f
170	F(TDPPTBFU)2	PC71BM	-5.2	-3.49	0.729	2.07	0.51	0.77	10.1039/c3ee24351f

171	SOHDT(TDPPTB FU)2	PC71B M	-5.19	-3.64	0.835	3	0.51	1.28	10.1039/c3ee24351f
172	Bdt-dpp	PC71B M	-5.15	-3.44	0.72	11.8 6	0.62	5.29	10.1021/am302896u
173	m1	PC61B M	-5.8	-3.83	0.88	5.5	0.64	3.1	10.1039/C5TA06501A
174	m2	PC61B M	-5.79	-3.77	0.8	11.4	0.6	5.5	10.1039/C5TA06501A
175	IDT-2DPP	PC71B M	-5.11	-3.32	0.88	8.53	0.37 6	2.82	10.1039/c3ta13816j
176	DDPP-TTAR	PC71B M	-5.12	-3.17	0.722 2	10.4	0.53 5	3.98	10.1039/C5TC01348H
177	DDPPâ€™-TTAR	PC71B M	-5.06	-3.28	0.597	1.58	0.51 4	0.43	10.1039/C5TC01348H
178	DPP2(PTA)	PC71B M	-5.1	-3.58	0.77	6.82	0.45 7	2.39	10.1021/am5006223
179	DPP2(NPTA)	PC71B M	-4.96	-3.67	0.71	10.6 9	0.48 3	3.69	10.1021/am5006223
180	PCLDPP	PC71B M	-5.62	-3.74	0.89	9.7	0.56	4.8	10.1039/C5TA10430K
181	BDPT-2BT	PC71B M	-5.38	-3.48	0.84	5.08	0.51 2	2.18	10.1039/C5CP05474E
182	BDPT-2FBT	PC71B M	-5.55	-3.58	0.8	6.83	0.48 1	2.63	10.1039/C5CP05474E
183	BDPT-2DPP	PC71B M	-5.44	-3.69	0.84	9	0.52 4	3.97	10.1039/C5CP05474E
184	BDT(DPP-TTHEX)2	PC71B M	-5	-3.5	0.57	7.67	0.6	2.19	10.1039/C6RA01103A
185	BDT(DPP-TT)2	PC71B M	-5.16	-3.64	0.65	6.08	0.56	2.12	10.1039/C6RA01103A
186	F(DPP)2B2	PC71B M	-5.15	-3.61	0.68	13.3 9	0.45 3	5.03	10.1039/C4TA06035K
187	IDT-DPP	PC71B M	-5.21	-3.39	0.89	8.08	0.54	3.26	10.1002/aenm.201400879
188	T-DPP	PC71B M	-5.32	-3.8	0.66	8.16	0.43	2.3	10.1002/aenm.201400879
189	ING-DPP	PC71B M	-5.24	-3.7	0.66	8.47	0.4	2.1	10.1002/aenm.201400879
190	DPA(DPPBDT)2	PC71B M	-5.08	-3.6	0.62	15.4 3	0.58 2	5.61	10.1002/aenm.201500059
191	NBU(DPPBDT)2	PC71B M	-5.13	-3.63	0.66	8.23	0.56 2	3.09	10.1002/aenm.201500059
192	TPA-DPP-BDT	PC61B M	-5.16	-3.6	0.67	11.4	0.53 2	4.04	10.1016/j.orgel.2014.07.020
193	TPA-DPP-3T	PC71B M	-5.12	-3.37	0.68	8.58	0.48	2.8	10.1016/j.orgel.2014.07.020
194	DPP2Py	PC71B M	-5.25	-3.49	0.85	11.1 3	0.60 1	5.67	10.1039/C6TA00416D
195	THDPP2PY	PC71B M	-5.17	-3.47	0.66	15.3 5	0.57 9	5.88	10.1039/C6TA00416D
196	TPADPP2PY	PC71B M	-5.03	-3.37	0.62	10.7 7	0.63 9	4.26	10.1039/C6TA00416D
197	PH(DPP-Th)2	PCBM	-5.27	-3.34	0.65	5.63	0.66	2.41	10.1016/j.dyepig.2015.10.00 6
198	PH(DPP-PY)2	PC71B M	-5.3	-3.16	0.85	10.5 5	0.39	3.22	10.1016/j.dyepig.2015.10.00 6
199	TPAKP-3	PC71B M	-5.15	-3.6	0.66	7.92	0.34 7	1.81	10.1039/c2jm16760c
200	TPA-T-DPP	PC71B M	-5.13	-3.32	0.81	10.0 6	0.36 2	2.95	10.1039/c3ta10640c

201	Tc12	PC71BM	-5.6	-3.84	0.75	10.17	0.58	4.39	10.1021/acs.jpcc.5b10064
202	(P-DPP)3TPA	PC61BM	-5.11	-3.39	0.72	7.94	0.52	2.98	10.1021/am302623k
203	4-BU[-DPP)3TPA	PC61BM	-5.09	-3.41	0.8	5.83	0.42	1.98	10.1021/am302623k
204	TPA(DPP-PN)3	PC71BM	-4.97	-3.46	0.8	8.95	0.51	3.67	10.1039/C4TA03688C
205	TPA(DPPT2)2	PC71BM	-4.58	-2.65	0.64	9.48	0.63	3.69	10.1039/c5ra07383a
206	3D-B-T-P	PC71BM	-5.14	-3.6	0.77	7.69	0.59	3.41	10.1002/asia.201300371
207	5A	PC71BM	-5.4	-3.75	0.89	8.98	0.606	4.8	10.1002/adma.201302007
208	5B	PC71BM	-5.2	-3.56	0.86	10.4	0.617	5.5	10.1002/adma.201302007
209	MV143	PC71BM	-5.53	-3.49	1.08	13.87	0.61	9.14	10.1039/c8cc08329k
210	DTFBR	PC71BM	-5.54	-3.68	1.08	6.94	0.32	2.42	10.1039/c8qm00223a
211	BD-pPor	PC71BM	-5.39	-3.74	0.88	12.43	0.61	6.67	10.1039/c8ta01291a
212	BD-tPor	PC71BM	-5.36	-3.79	0.95	14.32	0.67	8.98	10.1039/c8ta01291a
213	SM1	PC71BM	-5.43	-3.31	0.765	13.53	0.62	6.4	10.1039/c8ta02699h
214	BDTTT-DPP	PC61BM	-5.04	-3.46	0.828	10.92	0.61	5.53	10.1039/c8tc00799c
215	BDTTVT-DPP	PC61BM	-5.07	-3.43	0.83	7.32	0.57	3.48	10.1039/c8tc00799c
216	DRC4TB	PC71BM	-5.25	-3.21	0.94	13.16	0.69	8.53	10.1039/c9tc04680a
217	Si-PO-2CN	PC71BM	-4.98	-3.15	0.701	12.54	0.64	5.6	10.1002/chem.201903599.
218	FG1	PC71BM	-5.35	-3.62	0.85	11.92	0.55	5.57	10.1039/d0tc00154f
219	FG2	PC71BM	-5.15	-3.55	0.78	14.94	0.6	6.99	10.1039/d0tc00154f
220	FG3	PC71BM	-5.39	-3.72	0.91	14.24	0.58	7.51	10.1039/d0tc00154f
221	FG4	PC71BM	-5.17	-3.69	0.78	16.38	0.66	8.43	10.1039/d0tc00154f
222	BDT-Qx	PC71BM	-5.34	-3.26	0.75	2.2	0.31	0.52	10.1080/15421406.2020.1741817
223	BDT-T-Qx	PC71BM	-5.35	-3.4	0.78	2.41	0.32	0.59	10.1080/15421406.2020.1741817
224	TPA(BT-CNC8)2	PC71BM	-5.4	-3.68	0.81	6.94	0.29	1.62	10.1088/2053-1591/aace70
